# Visualization for Departures from Symmetry with the Power-Divergence-Type Measure in Square Contingency Tables

**DOI:** 10.1017/psy.2025.10057

**Published:** 2025-11-03

**Authors:** Wataru Urasaki, Tomoyuki Nakagawa, Jun Tsuchida, Kouji Tahata

**Affiliations:** 1 Tokyo University of Science - Noda Campus, Japan; 2 Meisei University - Hino Campus, Japan; 3 Kyoto Women’s University, Japan

**Keywords:** correspondence analysis, measure of asymmetry, power-divergence, square contingency table, visualization

## Abstract

When the row and column variables consist of the same category in a two-way contingency table, it is called a square contingency table. Since square contingency tables have an association structure due to the concentration of observed values near the main diagonal, a primary objective is to examine symmetric relationships and transitions between variables. Various models and measures have been proposed to analyze these structures to understand the changes between two variables’ behavior at two-time points or cohorts. This is necessary for a detailed investigation of individual categories and their interrelationships, such as shifts in brand preferences. We propose a novel approach to correspondence analysis (CA) for evaluating departures from symmetry in square contingency tables with nominal categories, using a modified divergence statistic. This approach ensures that well-known divergence statistics can also be visualized and regardless of the divergence statistics used, the CA plot consists of two principal axes with equal contribution rates. Notably, the scaling of the departures from symmetry provided by the modified divergence statistic is independent of sample size, allowing for meaningful comparisons and unification of results across different tables. Confidence regions are also constructed to enhance the accuracy of the CA plot.

## 1 Introduction

Categorical variables, which share the same category classification, appear in various fields, including medicine, education, and social science, and have long been the subject of analysis. A table derived from the combination of the variables is known as a square contingency table. Numerous studies have analyzed contingency tables and evaluated the independence or association between variables, but recently, new methods have been proposed by Chatterjee (2021), Forcina & Kateri (2021), Kateri (2018), and Urasaki et al. (2024). In the square contingency table, the observed values are concentrated in the diagonal components and tend to decrease as they move away from the diagonal components. This feature clearly shows a strong association, making traditional analysis methods inappropriate. Therefore, research has increasingly focused on symmetry and the transition between variables has advanced in the context of square contingency table analysis. It is important to investigate how similar or transitional the variables are between the two-time points or cohorts, and explore the departure from symmetry is also of interest.

There are two well-known methodological approaches to the analysis of symmetry: model and measure. As for models, in addition to the symmetry test (Bowker, 1948), referred to as the symmetry (S) model in Goodman (1979) and Agresti (2010), the marginal homogeneity (MH) model (Stuart, 1955) and the quasi symmetry (QS) model (Caussinus, 1965) are well known, as are the recently proposed models with divergence by Kateri (2021) and Tahata (2022). The measure approach evaluates the degree of departure from the models within a fixed interval regardless of sample size. These features enable the quantification and comparison of the degrees of departure from the model for each contingency table observed across various factors, including confounding issues. As an example, Tomizawa et al. (1998) proposed a generalized divergence-type measure that guarantees that the degree of departure from the S model can be evaluated in the range of 0 to 1 by a power-divergence. (For more details on power-divergence statistics, see Cressie & Read (1984), Read & Cressie (1988).)

While many methods have been proposed to understand the overall structure of contingency tables, a method to understand the relationships among categories through visualization has existed for a long. This is called correspondence analysis (CA), proposed by Benzécri, which has aided the realization of sophisticated and easy-to-understand visualizations, enabling quick interpretation and understanding of data. Simple CA is still widely used and easily implemented using tools, such as SAS, R, and Python. It is a visualization based on Pearson’s chi-square statistic, described in detail by Beh & Lombardo (2014) and Greenacre (2017). Visualization based on the approximations of power-divergence statistics has also been proposed by Beh & Lombardo (2024b). Many proposals aim to understand relationships among categories of the contingency table based on independence or association evaluation methods. However, visualizations focusing on departures from symmetry have been proposed by Beh & Lombardo (2022, 2024a), Greenacre (2000), and Van der Heijden et al. (1989). In particular, Greenacre (2000) and Van der Heijden et al. (1989) provide a visualization approach based on singular value decomposition (SVD) applied to the skew-symmetric matrix 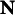 , which is obtained as the residual matrix between the asymmetric square matrix of observed proportions 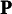 and the proportion matrix 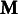 under the *S* model. Several studies have applied the SVD approach with Constantine & Gower (1978) and Tomizawa & Murata (1992) serving as examples. Similarly, Beh & Lombardo (2022, 2024a) also provide a visualization based on the SVD approach to a skew-symmetric matrix, but uses a skew-symmetric matrix derived from residuals based on power-divergence statistics. These proposals, which utilize the residuals, have the advantage of treating the power-divergence statistic as an index of departure from symmetry. Additionally, the principal coordinates and singular values obtained through the SVD can be directly linked to the statistic, allowing for a more concise and interpretable discussion.

In this study, we propose a methodological approach to visually provide the relationship between nominal categories based on the degree of departure from symmetry by the modified power-divergence statistics in two-way square contingency tables. This study, similar to Beh & Lombardo (2024a), provides a visualization of departures from symmetry based on the SVD of a residual matrix derived from power-divergence statistics. However, our proposal, which employs the modified power-divergence statistics, offers several advantages. A particularly significant advantage is that the scaling of departures from symmetry, quantified by Tomizawa et al.’s measure, is independent of sample size. This allows for meaningful comparisons and unification of CA plots across square contingency tables with different sample sizes. A detailed discussion on this point, including an analysis using two real datasets with different sample sizes, is provided in Section 6. Furthermore, unlike Beh & Lombardo (2024a), our approach avoids the second-order Taylor series approximation of power-divergence statistics and provides an exact CA framework without relying on approximations. We discuss the characteristics of CA in the context of symmetry about Tomizawa et al.’s measure, the construction of confidence regions for each category in the CA plot, and applications to real data. These contributions are expected to offer new insights into symmetry.

## 2 Analysis of symmetry

Consider an 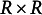 contingency table with nominal categories for the row variable *X* and the column variable *Y*. Let 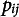 denote the probability that an observation will fall in the *i*th row and *j*th column of the table ( 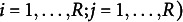 . Let 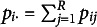 and 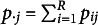 denote the marginal probabilities. Conversely, let 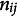 denote the observed frequency in the *i*th row and *j*th column of the table. The totals 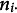 , 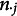 , and *n* are also denoted as 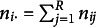 , 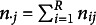 , and 
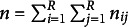
 , respectively. Furthermore, when analyzing symmetry using real data, it is common to assume a multinomial distribution for the 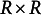 contingency table and to replace the cell probabilities 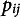 with their estimates 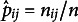 . Using this notation, we introduce Bowker’s test and Tomizawa et al.’s measure.

### 2.1 Bowker’s test statistic

Bowker’s test, for symmetry, may be undertaken by defining the S model with 

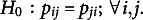

Under the null hypothesis 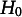 , Bowker’s 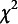 statistics is presented as follows: 

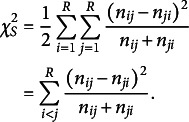

It follows a chi-squared distribution with 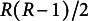 degrees of freedom. 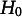 is rejected for high values of 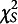 . Bowker’s test is a generalization of McNemar’s test (McNemar, 1947) for an 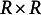 contingency table with 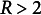 . Beh & Lombardo (2022) proposed a visualization of the degree of departures from symmetry based on 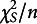 .

### 2.2 Tomizawa et al.’s power-divergence-type measure

When the S model does not hold by Bowker’s test, one can quantitatively evaluate the degree of departure from symmetry. In particular, Tomizawa et al. (1998) proposed the following power-divergence-type measure 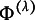 based on the power-divergence, to quantify departures from symmetry: 

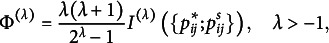

where 

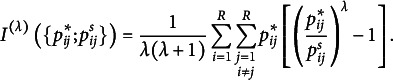

Assume that 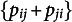 for 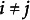 are all positive, and 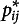 and 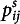 are defined as 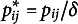 and 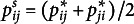 , with 
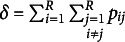
 . An interesting aspect of this measure, 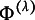 , is that it utilizes the conditional probability 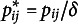 instead of 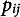 . This represents the probability of an observation falling into the ( 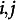 ) cell since it does not fall into the main diagonal cells. In the analysis of square contingency tables, the symmetry model does not impose any constraints on the cell probabilities along the main diagonal of the table. Therefore, Tomizawa (1994) proposed Pearson and KL divergence-type measures to quantify the departure from symmetry under the condition that an observation falls into a cell other than those on the main diagonal. As a result, the conditional probability 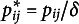 was directly used instead of 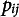 . Furthermore, 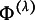 , proposed in Tomizawa et al. (1998), serves as a generalization of the measures introduced in Tomizawa (1994). Consequently, it adopts 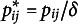 in its formulation.

The measure 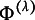 shares the following three properties for 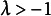 , similar to those of 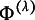 described in Tomizawa et al. (1998). Additionally, the parameter 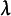 is determined by the user, and the case of 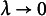 is treated as 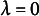 .

Theorem 2.1. The 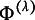 satisfies the following properties for all 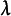 :

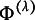
 must lie between 0 and 1.

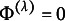
 if and only if there is a complete structure of symmetry, that is, 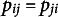 .

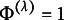
 if and only if there is a structure in which the degree of departure of symmetry is the largest, that is, 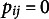 (then 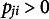 ) or 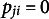 (then 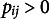 ).

When analyzing real data using the measure, the estimated value of Tomizawa et al.’s power-divergence-type measure, 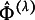 , is calculated using the plug-in estimators 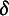 , 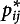 , and 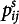 , where 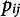 is replaced by 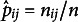 . We refer to 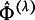 as the modified power-divergence statistics, because it is derived by normalizing the power-divergence statistics for symmetry such that its maximum value is 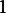 . In particular, when 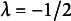 , 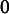 , 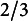 , and 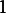 , the plug-in estimator 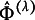 relates to famous divergence statistics with a special name, called Freeman–Tukey statistic, KL divergence statistic (log-likelihood statistic), Cressie–Read statistic, and Pearson’s divergence statistic, respectively. However, note that the measure by Tomizawa et al. (1998) is defined only for 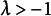 to ensure that its values remain between 0 and 1. Therefore, as a limitation, some important divergence statistics, such as the reverse KL divergence statistic ( 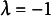 ) and the reverse Pearson’s divergence statistic ( 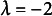 ), cannot be applied. The relationship between the values of the power-divergence statistic proposed by Cressie & Read (1984) and the estimates of the measure is detailed in Section 6 (Concluding Remarks) of Tomizawa et al. (1998).

The important point in this Section 2.2 is that the sample size *n* does not appear in the estimator 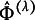 . Therefore, the major benefit of the estimator 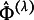 is that it can be quantified independently of the sample size, making it suitable for comparing the asymmetry of multiple contingency tables. While the 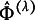 may seem a simple modification of the power-divergence statistic of Cressie & Read (1984) due to the adjustment by 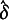 , its properties are significantly different, and this distinction is notable. Additionally, to ensure that the 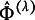 takes values between 0 and 1 independently on the sample size, the parameter is constrained by 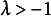 . Simple CA based on the estimator 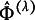 retains the same advantages and limitations, specifically its independence of sample size and the need to constrain the parameter 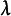 within a specific range to maintain this property.

## 3 Visualization and modified power-divergence statistics in simple CA

Visualization of relationships between categories in contingency tables enables rapid interpretation and understanding of data, even for non-experts. In particular, when examining the symmetry of nominal categories, it is important to analyze the similar or transitional relationship of categories between two-time points or cohorts. This section shows that the modified power-divergence statistics can be used to visualize the symmetric structures and interrelationships of individual categories.

### 3.1 SVD of matrices by modified power-divergence statistics

The estimator 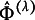 (and the measure 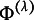 ) can also be defined as 

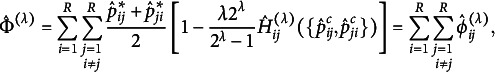

where 

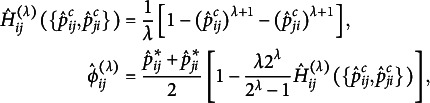

and 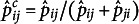 . The estimator 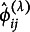 estimates a partial measure that quantifies the departure from symmetry for each ( 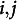 ) cell of the contingency table and is non-negative. Let us consider the 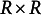 matrix 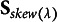 , constructed from the estimator 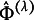 , with zero diagonal elements and the following ( 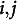 ) elements: 

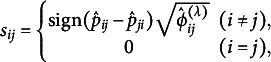

where 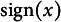 is a sign function defined as follows: 

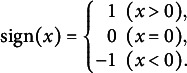

The matrix with such elements is called an anti-symmetric or skew-symmetric matrix, and 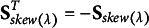 . The matrix 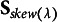 can also be interpreted as a residual matrix derived from the modified power-divergence statistic, scaled by the 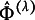 to ensure that it is not affected by sample size. Moreover, when the contingency table exhibits perfect symmetry, the matrix becomes a zero matrix. If symmetry exists only for certain categories, all elements in the rows and columns corresponding to those categories are zero. Using this matrix, the 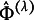 can be expressed as 

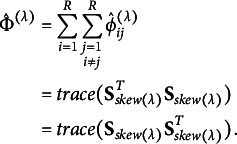

It indicates that 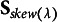 can reconstruct the 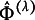 with information about the degree of departure from symmetry for each cell. The fact that such reconstruction is possible implies that, as discussed in Section 3.3, the total inertia in this study can be represented by 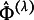 , just as in a standard simple CA where the total inertia is expressed as 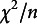 using the chi-square test statistic 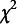 .

To visualize departures from symmetry, the matrices representing the principal coordinates of the row and column categories are obtained from the SVD of 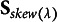 , that is, 

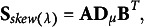

where 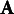 and 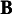 are 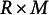 orthogonal matrices containing left and right singular vectors of 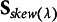 , respectively, and 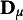 is 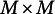 diagonal matrix with singular values 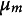 ( 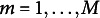 ). The singular values 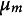 are also lined up in consecutive pairs of values, so that 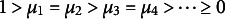 . Since 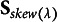 is a skew-symmetric matrix, *M* varies depending on the number of categories. Therefore, 

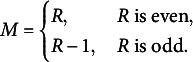

The SVD of the skew-symmetric matrix 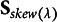 can also be expressed as 

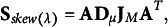

where 

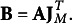




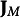
 is a block diagonal and orthogonal skew-symmetric matrix made up of 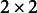 blocks 

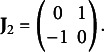

If *R* is odd, the matrix has a 0 in the final position. For a detailed description of features of the skew-symmetric matrix, see Gower (1977).

### 3.2 Principal coordinates and their characteristics

To obtain the principal coordinates of row and column categories using these matrices 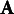 , 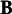 , and 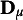 in the case of symmetry, we propose row and column coordinate matrices expressed as 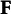 and 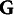 , respectively, defined as 

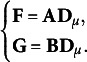

When defining 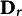 and 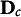 as diagonal matrices with 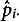 and 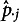 as their diagonal elements, the metric matrix 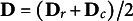 , used in Greenacre (2000) and Beh & Lombardo (2022, 2024a), is not used in the construction of 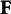 and 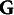 in this study. Note that the elements 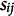 of 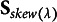 can be expressed in the following form based on the matrices 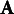 and 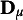 obtained via the SVD of 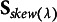 : 

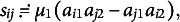

where 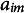 is the ( 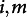 )th element of 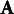 . From the above, the element 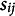 is given in terms of a triangle formed by the origin and the coordinate pairs of categories ( 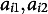 ) and ( 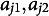 ). The expression 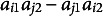 can be either positive or negative values. A positive value indicates that 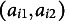 is positioned clockwise related to 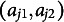 , while a negative value indicates a counterclockwise position. When 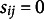 and 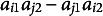 approaches zero, 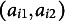 , 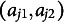 , and the origin appear to be collinear, though not perfectly aligned. If the area of the triangle is large, it can be interpreted as a greater departure from symmetry between category *i* and category *j*. The approach of using areas to visually represent asymmetry has been proposed in Corcuera & Giummolé (1998) and Chino (1990) and is considered an effective method. Focusing on this property, when the ( 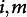 )th element 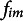 is defined as the row coordinate matrix 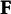 , the area of the triangle formed by the two-dimensional coordinates of the *i*th and *j*th row categories, 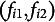 and 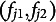 , along with the origin, is given as 

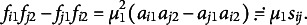

Although the overall area is scaled by 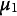 due to the absence of the metric 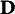 , this demonstrates that the areas derived from our constructed coordinate matrix allow for a simple and effective evaluation of individual 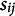 . Therefore, the visualization derived from the coordinate matrix **F** can provide an interpretation similar to that in Corcuera & Giummolé (1998) and Chino (1990). The same is true for the column coordinates.

Furthermore, the principal coordinates are also expressed as follows using 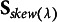 : 

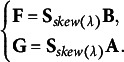

This representation implies that the principal coordinates vary depending on the parameter 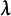 . However, since both the row and column spaces are based on an aggregation of 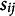 determined by the 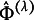 , they share the same departures from symmetry regardless of the value of 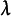 . Now, we consider 

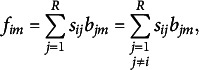

where 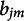 is the ( 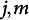 )th element of **B**. Note that 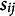 reflects the ( 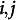 )th cell’s degree of departure from symmetry, 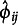 . Since 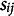 reflects the degree of departure from symmetry in the ( 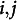 )th cell, represented by 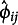 , it follows that a deviation of 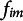 from zero indicates a departure from symmetry. Therefore, if the *i*th row category is located at the origin, this implies perfect symmetry between the *i*th row and all columns. In other words, for any *i*, 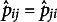 holds for all 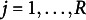 . Additionally, since 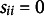 , 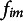 can be regarded as summarizing the departure from symmetry associated with the *i*th category. The same is also true for the column coordinates.

A property of these coordinates is that, since 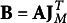 , the column coordinate matrix can be expressed in terms of the row coordinate matrix as 

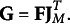

The fact that the column coordinate matrix can be expressed in this manner indicates that the row coordinate matrix effectively aggregates information related to departures from symmetry and is sufficient to represent information about column categories. Therefore, when constructing a CA plot using the method proposed in this study, plotting only the row coordinates can be considered sufficient. This plotting approach can also be observed in Greenacre (2000), which presents a CA plot derived from the CA of the skew-symmetric component. Since the relationship 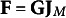 holds, the converse is also true, meaning that either row or column coordinates, and not both, can fully represent the asymmetry in the data. Additionally, since departures from symmetry capture changes between paired row and column categories, we can consider that the asymmetry information conveyed by row and column coordinates is essentially the same when no distinction is made between response and explanatory variables. In such cases, it is reasonable to plot only the row or column coordinates, as they sufficiently represent the degree of asymmetry without any loss of information.

### 3.3 Total inertia

When visualizing with the principal coordinates, we can quantify how much the coordinate axes in *m* dimensions ( 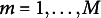 ) reflect the degree of departure from symmetry by calculating the total inertia of 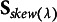 . The total inertia can be expressed as the sum of squares of the singular values, as follows: 

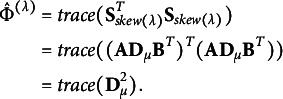

When constructing the CA plot, the principal inertia of the *m*th dimension is assumed to be 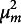 , to realize visualization up to the maximum *M* dimensions. The fact that the total inertia can be expressed as the sum of squares of the singular values determines the contribution ratio that indicates how much the coordinate axes of each dimension are reflected from the values of 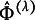 . Therefore, the contribution ratio of the *m*th dimension is calculated by 

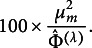

Visualization using the CA plot is ideally limited to a maximum of three dimensions due to the constraints of human cognitive capacity. Because of the limitation, we need to select any two dimensions, but in this case, it is appropriate to use the first and second dimensions. The singular values obtained from the skew-symmetric matrix are such that 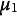 and 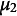 are the largest pairs, and the first and second dimensions are the best visually optimal representations reflecting the departures from symmetry.

### 3.4 Relationship between total inertia and principal coordinates

In the previous sections, we discussed how to construct the principal coordinates can be constructed for the row and column categories required to visualize departures from symmetry, and how to evaluate the extent to which asymmetry is reflected in the CA plot can be evaluated using total inertia. In this section, we briefly summarize the relationship between total inertia and principal coordinates.

While the total inertia of 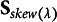 is expressed as the sum of squared singular values 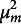 , it can also be represented using the row coordinate matrix 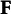 , as follows: 

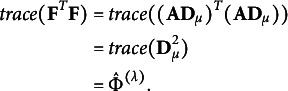

This shows that the row coordinate matrix 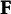 can reconstruct the estimator 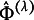 . From this expression, it also follows that when perfect symmetry holds in the contingency table, all row coordinates lie at the origin. A similar relationship holds for the column coordinate matrix as well, since 

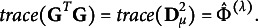

These formulations of total inertia also indicate that categories located farther from the origin correspond to greater departures from symmetry.

## 4 Confidence regions for individual categories

In Section 3, we demonstrated that visualization for departures from symmetry indicates complete symmetry when located at the origin, and the further a point is from the origin, the greater the departure from symmetry for each category. Therefore, while visualizing categories in terms of symmetry is important, understanding the interrelationships among categories is equally crucial. As for independence, Beh (2001) and Lebart et al. (1984) proposed confidence circles and Alzahrani et al. (2023) and Beh (2010) introduced confidence ellipses for each category based on the simple CA. Greenacre (2017) and Ringrose (1992, 1996) proposed constructing a non-circular confidence region using a convex hull by applying a bootstrap method. Greenacre (2017) also proposed a method for constructing confidence intervals using the delta method (see Agresti, 2012; Bishop et al., 2007). However, this approach has several limitations, including the assumption of independent random sampling and reduced approximation accuracy in small samples, which should be considered when using it. In this section, we also discuss the construction of the confidence regions in symmetry.

Let 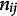 denote the observed frequency at the intersection of the *i*th row and *j*th column within the table. Assuming a multinomial distribution for the 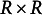 table and a significance level of 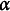 , typically taking values, such as 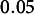 or 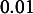 , the 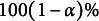 confidence region for the *i*th row category in the two-dimensional CA plot is expressed as follows: 

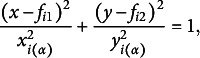

where the semi-axis lengths 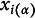 and 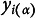 are 

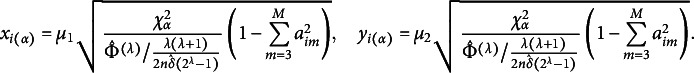

The 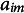 is the ( 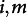 )th element of 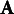 . 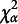 is the upper 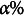 point of the chi-square distribution with 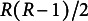 degrees of freedom. The proof for constructing the confidence region is given in Appendix 1. Since 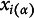 and 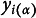 depend on significance level 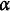 , lowering 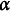 to increase the level of confidence results in an expansion of the confidence region. This indicates that the construction of such confidence intervals appropriately ensures the accuracy of the points. Note that the singular values 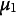 and 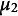 are equal, so the confidence regions are circular.

## 5 Numerical experiment

Consider Table 1 by Agresti (2019), from which the original data come from Grover & Srinivasan (1987). Table 1 shows the data on the first and second purchase choices for five brands of decaffeinated coffee. The symmetry of the row and column variables in such data suggests a balanced inflow of people who tend to choose different products regardless of the product they had initially selected. Therefore, departures from symmetry in each category indicate that for a given coffee brand, the number of new buyers and those who stopped buying are uneven. As it is important to investigate whether there is any difference in the inflow of people in each category and how many categories’ interrelationships are there, an analysis using our proposal is necessary. For the analysis, 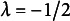 , 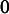 , 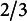 , and 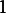 are applied to 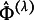 as parameters to visualize. Since 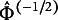 , 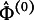 , 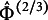 , and 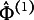 are based on the Freeman–Tukey, KL divergence, Cressie–Read, and Pearson divergence statistics, respectively, these estimators were used to construct the CA plots. Based on the relationship between row and column coordinates shown in Section 3, only row categories were plotted.

**Table 1:** Choice of first and second purchases of five brands of decaffeinated coffee

	Second purchase					
First purchase	High Pt	Taster’s	Sanka	Nescafé	Brim	Total
High point (HP)	93	17	44	7	10	171
Taster’s choice (TC)	9	46	11	0	9	75
Sanka (SA)	17	11	155	9	12	204
Nescafé (NE)	6	4	9	15	2	36
Brim (BR)	10	4	12	2	27	55
Total	135	82	231	33	60	541

**Figure 1:**
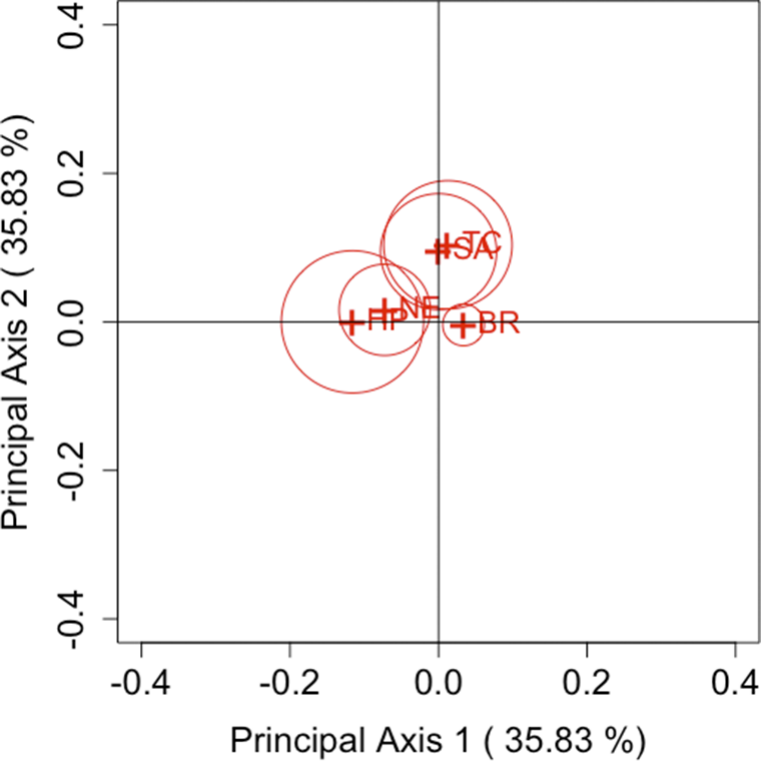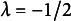 (Freeman–Tukey statistic).

**Figure 2:**
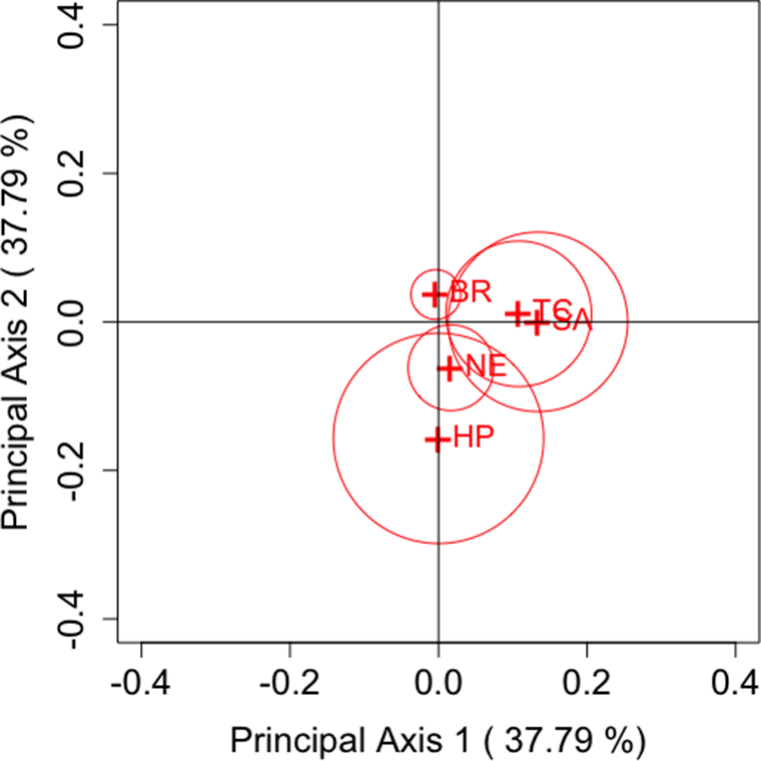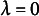 (KL divergence statistic).

**Figure 3:**
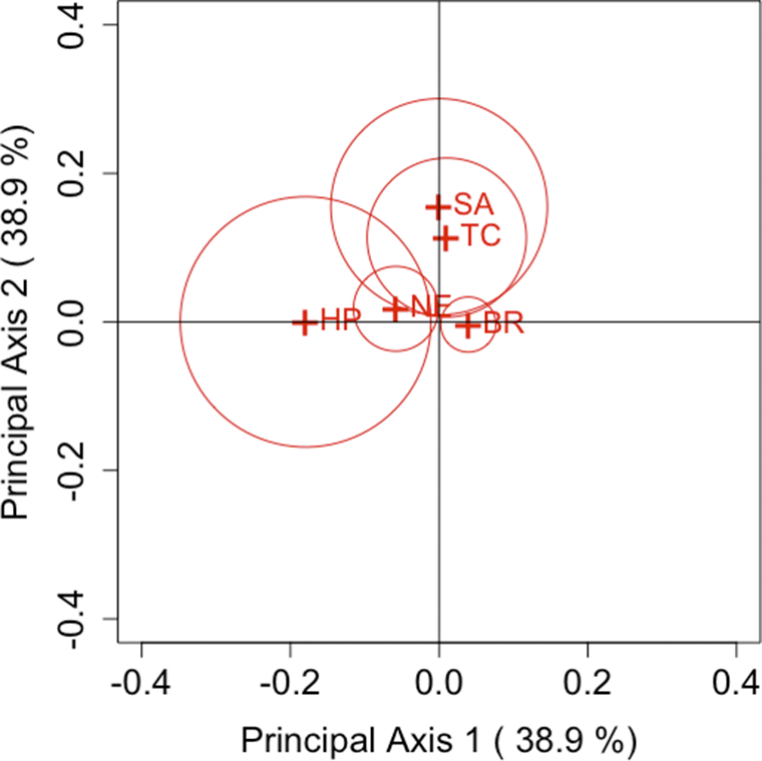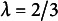 (Cressie–Read statistic).

**Figure 4:**
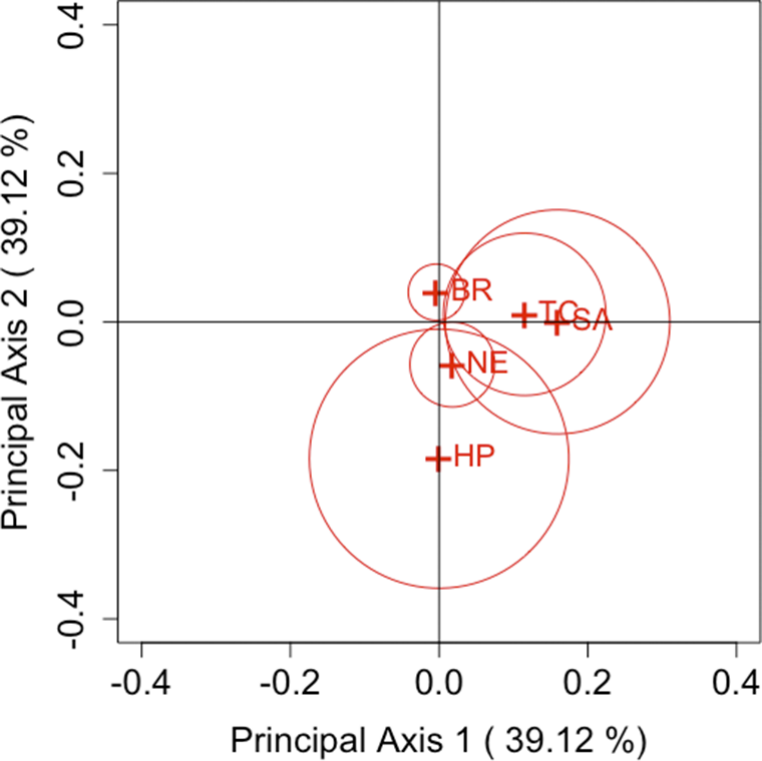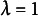 (Pearson’s divergence statistic).

**Table 2:** Example for the values of 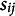 by 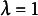

	Second purchase				
First purchase	High Pt	Taster’s	Sanka	Nescafé	Brim
High point (HP)	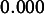	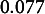	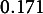	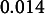	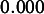
Taster’s choice (TC)	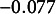	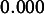	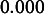	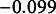	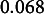
Sanka (SA)	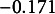	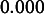	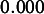	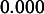	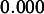
Nescafé (NE)	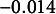	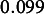	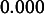	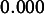	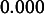
Brim (BR)	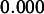	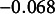	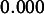	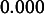	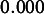

*Note*: The values of 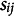 are approximately related to the area of the triangle formed by the paired categories *i* and *j*, and the origin.

Figures 1–4 show the results of analysis with each parameter and Table 2 shows an example for the values of 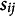 by 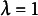 . As can be seen from these figures, Brim is located close to the origin for all parameters, while High Point is located far from the origin for many parameters. Other brands can also be seen as located far from the origin. Therefore, the following considerations can be made for each brand.

- Brim has only a slight departure from symmetry, indicating a small difference between the first and second purchase choices related to Brim. Therefore, while there is an observable influx of consumers purchasing Brim’s decaffeinated coffee, it can be assessed as relatively small compared with other brands.
- High Point has a larger departure from symmetry than other brands, indicating a considerable difference between the first and second purchase choices related to High Point. Thus, it can be inferred that High Point’s decaffeinated coffee may be more susceptible to influences such as purchase preferences compared to other brands.
- For Taster’s Choice, Nescafé, and Sanka, some changes are observed in the purchase choices.

Interestingly, at first glance, when comparing the number of decaffeinated coffee purchasers for each brand in the first and second purchases in Table 1, it may appear that Nescafé exhibits less variation. However, since the evaluation is based on coordinates that integrate the asymmetry of each ( 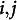 ) cell, Brim is actually assessed as having less variation. Providing a visualization that reflects individual departures, as demonstrated by these results, is considered highly useful when analyzing square contingency tables with a large number of categories. Additionally, we can also confirm that the confidence regions of all brands do not include the origin for each parameter 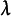 . Regardless of the differences in measurement methods for each famous divergence statistic, significant departures from symmetry are observed across all brands with sufficient accuracy.

Next, we focus on the positioning of each category’s point in the CA plots. For each parameter, it can be observed that Taster’s Choice, Sanka, and Brim, which are located in the positive direction of principal axis 1 or 2, show an increase in the total number of purchases in the second selection compared to the first. By contrast, High Point and Nescafé, positioned in the negative direction, show a decrease in purchases. These results suggest that the primary axes reflect the overall changes in the number of buyers for each brand. Another important aspect, as discussed in Section 3.2, is that the area of the triangle formed by the origin and the coordinates of any two categories *i* and *j*, 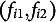 and 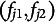 , reflects the departure from symmetry represented by the approximation of 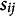 . Therefore, it can be evaluated that the largest departure occurs between High Point and Sanka, followed by the departure between High Point and Taster’s Choice. This can also be seen in Table 2. However, it should be noted that these areas are based on approximated values of 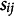 . For example, in the case of High Point and Brim, the areas may appear nonzero despite 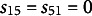 . Thus, caution is required when interpreting the areas visually.

Our proposed method effectively visualizes these relationships, as demonstrated in this analysis. Although such insights can be obtained by carefully examining the cell frequencies for each category pair in a two-way contingency table, our approach offers a clear advantage by enabling easy visual identification of patterns on the CA plot within the framework generalized by the parameter 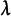 . This advantage becomes more pronounced as the number of categories increases, offering a concise evaluation of the symmetry relationships between categories.

Remark 5.1. (Brief guidelines for selecting the parameter 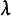 to be applied to the modified power-divergence statistic) It is important to provide theoretical guarantees for various divergence statistics within the range of the parameter 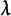 by utilizing the power-divergence statistic generalized by 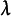 . However, in practical data analysis, we need to select specific parameter values, considering various factors, such as data and the ease of interpretation. Although selecting the optimal parameters remains one of the challenges to be addressed in our study, no mathematically valid method has been proposed until now. One practical method for parameter selection is to determine the parameters based on the user’s desired divergence statistic, considering the background of the data and aligning them with the analytical methods used in the user’s field and the structure of the data being analyzed. Another possible approach is to select parameters that maximize the percentage of total inertia in two dimensions in the CA plot, a method introduced by Cuadras & Cuadras (2006). However, even in such a method, it is unclear from what point of view the divergence statistic given by the selected parameters evaluates the departures from symmetry. Therefore, the user should adopt the divergence statistics that have properties suitable for an analytical purpose. If that is not possible, it would be better to select several well-known divergence statistics or examine with different values of various parameters in an exploratory manner.

## 6 Discussion: Why use a modified power-divergence statistics?

In Section 3, we proposed a novel approach to visualize the asymmetric relationship between nominal categories using the modified power-divergence statistics, 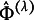 , focusing on departures from symmetry. Unlike the traditional approaches, our approach defines a scale for departures from symmetry that remains constant regardless of sample size. This is the key distinguishing feature of our approach.

Consider two 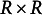 square contingency tables with sample sizes 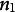 and 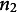 , respectively. In the method by Beh & Lombardo (2022, 2024a), if 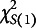 and 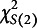 are the test statistics for the two tables, CA plots of the two can be created based on 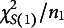 and 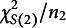 . However, note that because the scaling differs by 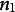 and 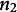 , comparing the two CA plots is not straightforward. Therefore, when visualizing multiple contingency tables, special procedures, such as Multiple CA or Joint CA, must be used. Detailed explanations of Multiple CA and Joint CA are available in Beh & Lombardo (2014) and Greenacre (2017). Proposals for comparing and visualizing categories by analyzing sum and difference components of several tables have also been made, such as in Greenacre (2003). However, Greenacre (2003) pointed out that, for CA, unless the sample sizes match, the differences in the patterns of category characteristics between tables are primarily influenced by the differences in sample size.

Let us reconsider our approach using the modified power-divergence statistics. As discussed in Section 2.2, the measure is independent of sample size, making it suitable for comparison. Therefore, by using the estimator 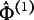 as an example, we demonstrate that it is possible to visually analyze both the sum and difference components of the tables.

### 6.1 Approach for matched square contingency tables

For the two 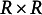 square contingency tables, let the skew-symmetric matrices constructed by our proposed method be 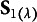 and 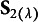 . We assume that these two matrices are constructed using the same value of parameter 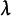 . The SVDs of the sum 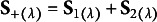 and the difference 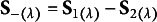 can be derived from the SVD of the following block matrix: 

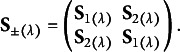

Suppose that the SVDs of 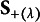 and 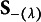 are, respectively, 

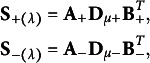

where 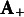 , 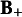 , 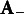 , and 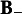 are 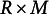 orthogonal matrices containing the left and right singular vectors of 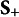 and 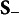 , respectively. Additionally, 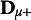 and 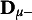 are diagonal matrices of the singular values 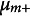 and 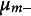 ( 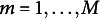 ) in each case. Note that 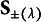 , 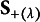 , and 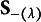 also become skew-symmetric matrices. Then, the SVD of 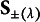 can be expressed as 

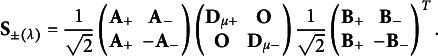

The details of the SVD are outlined in Greenacre (2003). Notably, when performing the SVD of this block matrix, the matrices obtained from the SVDs of 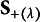 and 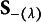 do not appear separated. Instead, they are interleaved according to the descending order of the values of 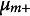 and 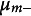 . The sum and difference of two squared contingency tables can be obtained by performing SVD for each case, without the need to construct a block matrix. However, using this approach allows us to achieve optimal visualization by relying solely on the SVD of a single skew-symmetric matrix. Additionally, this method can be extended to more than two matched matrices while maintaining the skew-symmetric structure.

### 6.2 Brief example

Consider Table 3 as a brief analysis example. Table 3 is taken from Agresti (1993), with the original data obtained from the 1989 General Social Survey conducted by the National Opinion Research Center at the University of Chicago. Subjects in the sample group were asked their opinion on (i) early teens (age 14–16) having sex relations before marriage, and (ii) a man and a woman having sex relations before marriage. The response scales of premarital and extramarital sex were (1) always wrong, (2) almost always wrong, (3) wrong only sometimes, and (4) not wrong at all. The analysis of the departures from symmetry in Table 3 refers to exploring the degree of disagreement between views on premarital and extramarital sex. Table 4 shows the results of the analysis on the sum and difference in opinions between early teens and the before marriage group.

The results of the SVD of the block matrix and principal coordinates are given in Table 4 by using the 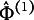 . Note that the singular values 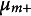 and 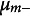 ( 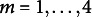 ) of the skew-symmetric matrix related to the sum and difference components are described as follows: 

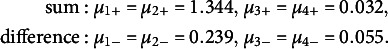

**Figure 5:**
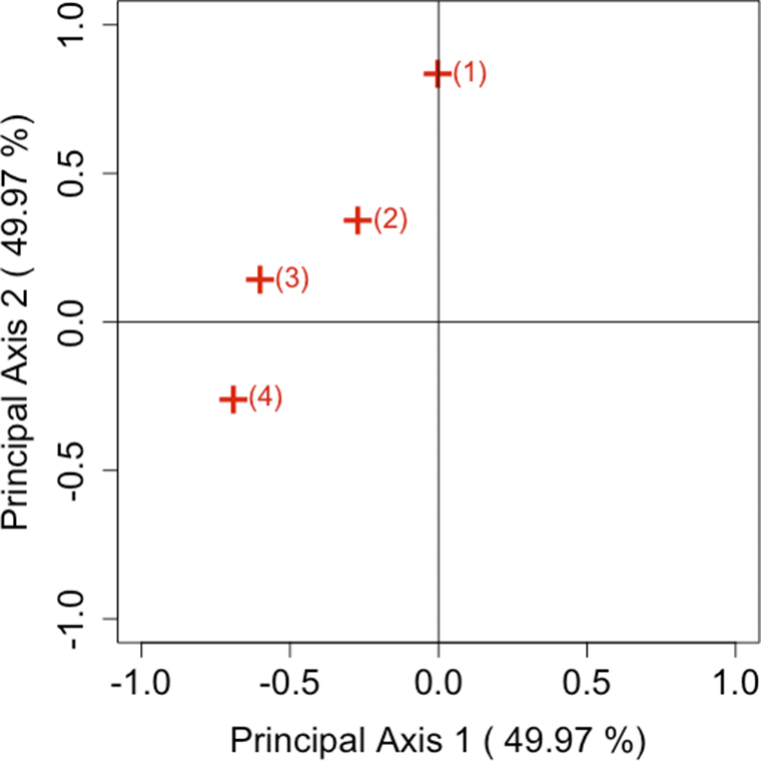
Visualization of the sum component of the response scales with 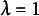 .

**Figure 6:**
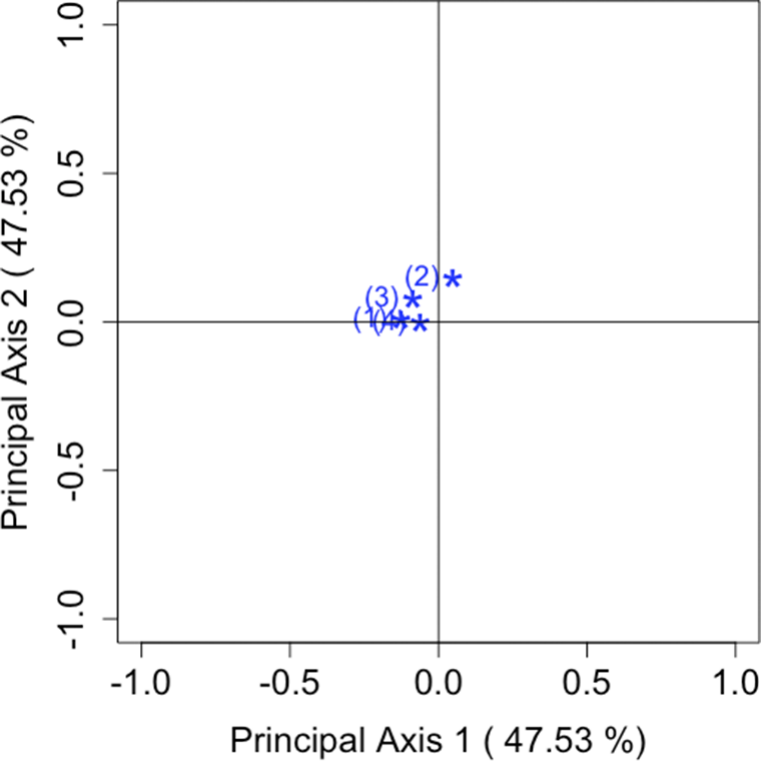
Visualization of the difference component of the response scales with 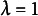 .

From these values, dimensions 1, 2, 7, and 8 correspond to the sum component, and dimensions 3, 4, 5, and 6 correspond to the difference. Therefore, the CA plot using dimensions 1 and 2 visualizes the sum component, while the plot using dimensions 3 and 4 visualizes the difference component, as illustrated in Figures 5 and 6. In addition, Table 5 shows the values of the element of 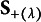 and 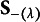 by 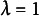 . The values are approximately related to the area of the triangle formed by the paired categories *i* and *j*, and the origin in Figures 5 and 6. As a brief example, we used 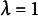 to create the CA plots, and the choice of the parameter 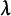 follows the same reasoning as described in Remark 5.1. However, when interpreting the plots in terms of inertia, care must be taken regarding whether the focus is on the sum component, the difference component, or both. The key observation in Figures 5 and 6 is the differing interpretation of the origin in each plot. Figure 5 shows the overall opinions for two sample groups, with points near the origin indicating that there is no departure from symmetry in the two groups. Conversely, Figure 6 presents the differences between the two sample groups, where points near the origin reflect that the two groups held similar views.

**Table 3:** Opinions about teenage sex, premarital sex, and extramarital sex from 1989 General Social Survey, with categories: (1) always wrong; (2) almost always wrong; (3) wrong only sometimes; and (4) not wrong at all

	Extramarital sex				
Premarital sex	1	2	3	4	Total
(i) Early teens					
(1)	140	1	0	0	141
(2)	30	3	1	0	34
(3)	66	4	2	0	72
(4)	83	15	10	1	109
Total	319	23	13	1	356
(ii) Before marriage					
(1)	3	1	0	0	4
(2)	3	1	1	0	5
(3)	15	8	0	0	23
(4)	23	8	7	0	38
Total	44	18	8	0	70

**Table 4:** SVD of 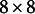 block matrix and principal coordinates

Dimension	1	2	3	4	5	6	7	8
Singular values								
	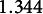	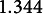	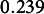	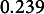	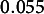	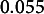	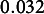	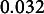
Right singular vectors								
First block	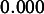	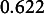	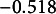	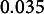	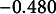	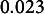	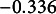	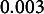
	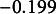	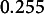	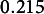	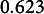	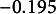	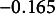	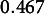	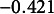
	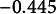	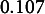	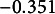	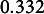	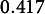	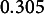	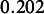	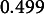
	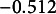	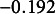	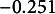	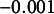	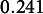	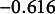	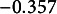	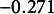
Second block	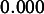	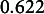	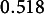	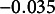	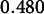	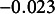	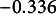	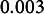
	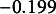	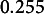	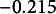	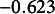	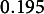	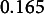	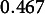	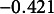
	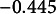	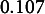	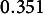	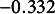	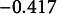	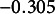	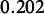	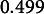
	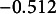	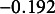	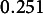	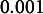	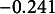	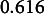	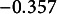	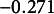
Left singular vectors								
First block	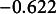	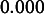	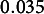	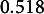	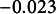	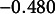	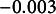	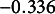
	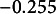	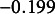	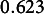	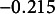	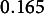	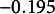	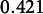	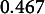
	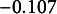	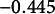	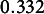	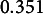	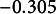	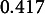	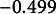	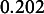
	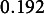	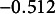	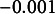	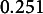	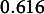	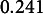	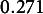	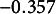
Second block	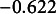	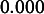	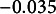	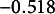	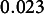	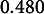	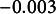	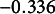
	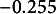	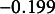	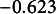	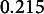	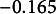	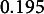	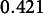	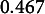
	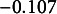	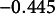	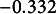	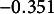	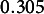	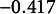	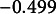	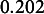
	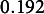	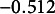	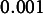	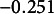	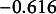	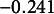	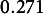	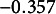
Principal coordinate by right singular vector								
First block	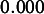	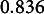	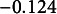	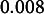	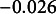	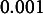	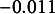	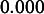
	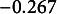	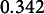	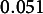	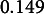	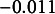	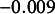	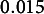	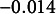
	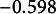	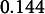	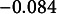	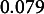	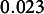	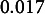	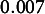	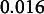
	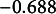	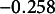	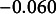	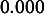	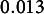	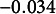	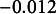	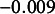
Second block	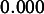	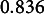	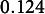	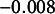	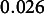	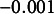	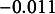	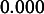
	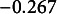	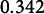	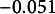	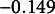	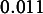	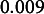	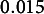	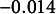
	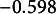	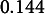	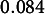	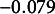	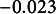	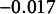	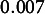	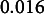
	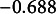	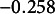	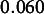	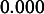	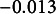	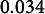	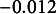	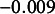
Principal coordinate by left singular vector								
First block	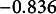	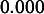	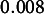	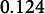	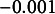	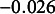	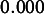	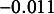
	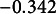	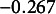	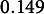	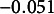	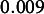	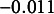	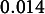	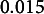
	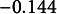	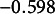	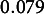	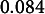	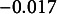	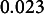	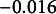	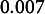
	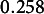	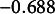	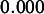	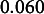	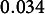	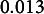	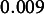	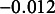
Second block	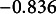	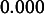	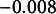	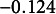	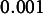	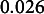	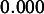	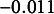
	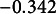	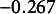	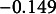	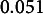	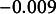	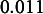	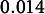	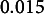
	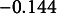	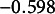	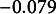	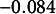	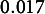	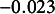	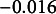	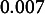
	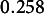	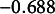	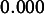	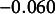	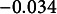	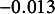	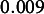	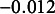

**Table 5:** The values of the element of 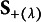 and 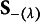 by 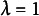

	Extramarital sex			
Premarital sex	1	2	3	4
Elements of 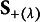				
(1)	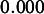	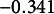	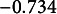	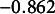
(2)	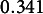	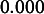	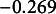	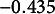
(3)	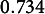	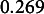	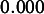	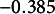
(4)	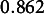	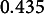	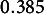	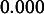
Elements of 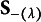				
(1)	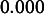	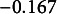	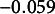	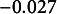
(2)	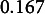	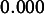	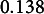	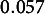
(3)	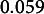	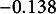	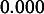	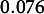
(4)	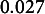	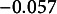	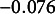	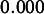

*Note*: The values are approximately related to the area of the triangle formed by the paired categories *i* and *j*, and the origin in Figures 5 and 6.

When considering the placement of the categories in the two CA plots, it becomes apparent that in Figure 5, all categories are situated far from the origin, while in Figure 6, they are located near the origin. These results suggest that, irrespective of whether the group is “early teen” or “before marriage” there is a disagreement in attitudes toward premarital and extramarital sex, but the opinions of the two groups remain consistent across all response scales. These considerations are apparent even from simply reviewing Table 3(i) and (ii). Despite a five-fold difference in sample sizes between Table 3(i) and (ii), our method effectively visualizes both the sum and difference components of asymmetry for each category pair. The ability to derive the same insight from both tables demonstrates a clear advantage of the proposed approach.

## 7 Conclusion

In this study, we propose a new visualization method for departures from symmetry with the modified power-divergence statistics, 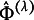 , in square contingency tables. Our method ensures that visualizations based on well-known and interpretable divergence statistics, such as the Freeman–Tukey statistic, KL divergence statistic, Cressie–Read statistic, and Pearson’s divergence statistic, are provided. Unlike the visualization approaches proposed by Constantine & Gower (1978) and Greenacre (2000), which apply SVD to a skew-symmetric matrix obtained from the decomposition of the observed matrix, our method is based on SVD applied to a skew-symmetric matrix derived from the estimator 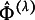 . As a result, the principal coordinates and singular values obtained through SVD are directly linked to the divergence statistics. This ensures that our approach can evaluate departures from symmetry using various modified power-divergence statistics for symmetry. Additionally, our method allows individual departures from symmetry 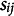 to be represented by the area of the triangle formed by the origin and the two plotted points, offering an interpretability of the visual approach advocated by Constantine & Gower (1978).

The evaluation of departures from symmetry in square contingency tables has been proposed by Beh & Lombardo (2024a) through the use of generalized statistics based on the power-divergence. While both our method and that of Beh & Lombardo (2024a) provide visualizations based on sufficiently generalized divergence statistics, several differences exist. A major difference in our approach, an advantage over Beh & Lombardo (2024a), is that the degree of departures from symmetry derived from the 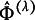 is independent on the sample size *n*. Since the 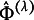 is suitable for comparing degrees of departure across multiple contingency tables, the analysis presented in Section 6 is feasible. Although the parameter 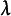 is restricted to values greater than 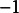 , our approach utilizes the exact power-divergence statistics without a second-order approximation. This allows for the construction of a skew-symmetric matrix and allows for effective visualization of departures from symmetry. Therefore, the features of our proposed method described above are essential for effectively analyzing departures from symmetry. We believe that these could provide new insights into symmetry.

## A Appendix. Proof of confidence region

Note that the confidence region was derived based on the proposal by Beh (2010). Proof. Let  be the (  )th cell element (  ) for  orthogonal matrix  with left singular vector. From the matrix **A**,

so that

holds. Considering the definition of the coordinates of the *i*th row category, it can also be expressed as follows:

where  and  are the (  ) and (  )th elements of the matrix **F** of row coordinates. Thus,

is obtained. This equation shows an ellipse centered at (  ,  ) whose major and minor radii are

and

respectively, on the two-dimensional CA plot. Similarly, we can obtain an elliptic formula for the *j*th column category. To construct a  confidence region for the *i*th row category, consider the squared singular value  of dimension *m* and the total inertia expressed by  . Assume that the upper  point of a chi-square distribution with  degrees of freedom is  and the singular value at  is  . Then, since the following power-divergence statistics

follows a chi-squared distribution, one can infer that

and

are obtained. Therefore, by replacing  and  in  and  with  and  , the  confidence region for the *i*th row category around (  ,  ) is given by

where

## References

[r1] Agresti, A. (1993). Computing conditional maximum likelihood estimates for generalized Rasch models using simple loglinear models with diagonals parameters. Scandinavian Journal of Statistics, 20(1), 63–71.

[r2] Agresti, A. (2010). Analysis of ordinal categorical data (Vol. 656). John Wiley & Sons.

[r3] Agresti, A. (2012). Categorical data analysis (Vol. 792). John Wiley & Sons.

[r4] Agresti, A. (2019). An introduction to categorical data analysis. John Wiley & Sons.

[r5] Alzahrani, A. A. , Beh, E. J. , & Stojanovski, E. (2023). Confidence regions for simple correspondence analysis using the Cressie-Read family of divergence statistics. Electronic Journal of Applied Statistical Analysis, 16(2), 423–448.

[r6] Beh, E. J. (2001). Confidence circles for correspondence analysis using orthogonal polynomials. Journal of Applied Mathematics and Decision Sciences, 5(1), 35–45.

[r7] Beh, E. J. (2010). Elliptical confidence regions for simple correspondence analysis. Journal of Statistical Planning and Inference, 140(9), 2582–2588.

[r8] Beh, E. J. , & Lombardo, R. (2014). Correspondence analysis: Theory, practice and new strategies. John Wiley & Sons.

[r9] Beh, E. J. , & Lombardo, R. (2022). Visualising departures from symmetry and Bowker’s *X*^2^ statistic. Symmetry, 14(6), 1103.

[r10] Beh, E. J. , & Lombardo, R. (2024a). Correspondence analysis for assessing departures from perfect symmetry using the Cressie–Read family of divergence statistics. Symmetry, 16(7), 830.

[r11] Beh, E. J. , & Lombardo, R. (2024b). Correspondence analysis using the Cressie–Read family of divergence statistics. International Statistical Review, 92(1), 17–42.

[r12] Bishop, Y. M. , Fienberg, S. E. , & Holland, P. W. (2007). Discrete multivariate analysis: Theory and practice. Springer Science & Business Media.

[r13] Bowker, A. H. (1948). A test for symmetry in contingency tables. Journal of the American Statistical Association, 43(244), 572–574. 18123073 10.1080/01621459.1948.10483284

[r14] Caussinus, H. (1965). Contribution à l’analyse statistique des tableaux de corrélation. Annales de la Faculté des sciences de l’Université de Toulouse, 29, 77–183.

[r15] Chatterjee, S. (2021). A new coefficient of correlation. Journal of the American Statistical Association, 116(536), 2009–2022.

[r16] Chino, N. (1990). A generalized inner product model for the analysis of asymmetry. Behaviormetrika, 17, 25–46.

[r17] Constantine, A. , & Gower, J. C. (1978). Graphical representation of asymmetric matrices. Journal of the Royal Statistical Society: Series C (Applied Statistics), 27(3), 297–304.

[r18] Corcuera, J. M. , & Giummolé, F. (1998). A characterization of monotone and regular divergences. Annals of the Institute of Statistical Mathematics, 50(3), 433–450.

[r19] Cressie, N. , & Read, T. R. (1984). Multinomial goodness-of-fit tests. Journal of the Royal Statistical Society: Series B (Methodological), 46(3), 440–464.

[r20] Cuadras, C. M. , & Cuadras, D. (2006). A parametric approach to correspondence analysis. Linear Algebra and its Applications, 417(1), 64–74.

[r21] Forcina, A. , & Kateri, M. (2021). A new general class of RC association models: Estimation and main properties. Journal of Multivariate Analysis, 184, 104741.

[r22] Goodman, L. A. (1979). Multiplicative models for square contingency tables with ordered categories. Biometrika, 66(3), 413–418.

[r23] Gower, J. C. (1977). The analysis of asymmetry and orthogonality. In J. R. Barra, F. Brodeau, G. Romer, & B. van Cutsem (Eds.), Recent Developments in Statistics, 109–123. Amsterdam: North-Holland.

[r24] Greenacre, M. (2000). Correspondence analysis of square asymmetric matrices. Journal of the Royal Statistical Society Series C: Applied Statistics, 49(3), 297–310.

[r25] Greenacre, M. (2003). Singular value decomposition of matched matrices. Journal of Applied Statistics, 30(10), 1101–1113.

[r26] Greenacre, M. (2017). Correspondence analysis in practice. Chapman; Hall/CRC.

[r27] Grover, R. , & Srinivasan, V. (1987). A simultaneous approach to market segmentation and market structuring. Journal of Marketing Research, 24(2), 139–153.

[r28] Kateri, M. (2018). φ-Divergence in contingency table analysis. Entropy, 20(5), 324. 33265414 10.3390/e20050324 PMC7512843

[r29] Kateri, M. (2021). Families of generalized quasisymmetry models: A φ-divergence approach. Symmetry, 13(12), 2297.

[r30] Lebart, L. , Morineau, A. , & Warwick, K. M. (1984). Multivariate descriptive statistical analysis. John Wiley & Sons, Inc.

[r31] McNemar, Q. (1947). Note on the sampling error of the difference between correlated proportions or percentages. Psychometrika, 12(2), 153–157. 20254758 10.1007/BF02295996

[r32] Read, T. R. , & Cressie, N. A. (1988). Goodness-of-fit statistics for discrete multivariate data. Springer Science & Business Media.

[r33] Ringrose, T. J. (1992). Bootstrapping and correspondence analysis in archaeology. Journal of Archaeological Science, 19(6), 615–629.

[r34] Ringrose, T. J. (1996). Alternative confidence regions for canonical variate analysis. Biometrika, 83(3), 575–587.

[r35] Stuart, A. (1955). A test for homogeneity of the marginal distributions in a two-way classification. Biometrika, 42(3–4), 412–416.

[r36] Tahata, K. (2022). Advances in quasi-symmetry for square contingency tables. Symmetry, 14(5), 1051.

[r37] Tomizawa, S. (1994). Two kinds of measures of departure from symmetry in square contingency tables having nominal categories. Statistica Sinica, 4(1), 325–334.

[r38] Tomizawa, S. , & Murata, M. (1992). Gauss discrepancy type measure of degree of residuals from symmetry for square contingency tables. Journal of the Korean Statistical Society, 21(1), 59–69.

[r39] Tomizawa, S. , Seo, T. , & Yamamoto, H. (1998). Power-divergence-type measure of departure from symmetry for square contingency tables that have nominal categories. Journal of Applied Statistics, 25(3), 387–398.

[r40] Urasaki, W. , Nakagawa, T. , Momozaki, T. , & Tomizawa, S. (2024). Generalized Cramér’s coefficient via f-divergence for contingency tables. Advances in Data Analysis and Classification, 18(4), 893–910.

[r41] Van der Heijden, P. G. , De Falguerolles, A. , & De Leeuw, J. (1989). A combined approach to contingency table analysis using correspondence analysis and loglinear analysis. Journal of the Royal Statistical Society: Series C (Applied Statistics), 38(2), 249–273.

